# Perceived Determinants of Disclosing Suicide Loss: A Qualitative Study Among Women

**DOI:** 10.1027/0227-5910/a000891

**Published:** 2022-11-29

**Authors:** Nathalie Oexle, Nadja Puschner, Nicole Votruba, Nicolas Rüsch, Lea Mayer

**Affiliations:** ^1^ Department of Psychiatry II, University of Ulm and BKH Günzburg, Ulm, Germany; ^2^ Nuffield Department of Women's & Reproductive Health, University of Oxford, UK; ^3^ Centre for Implementation Science and Centre for Global Mental Health, Institute of Psychiatry, Psychology and Neuroscience, King’s College London, UK

**Keywords:** suicide loss, disclosure, secrecy, grief, qualitative

## Abstract

**Abstract:** *Background:* People who lost a loved one to suicide (i.e., suicide loss survivors, SLS) often struggle to talk about their experiences. However, previous studies suggest beneficial effects of disclosure among this group. *Aims:* This study aimed to identify determinants of disclosing suicide loss. *Method:* We conducted qualitative interviews with 22 female SLS focusing on determinants of disclosing suicide loss. Interviews were transcribed and analyzed using qualitative content analysis. *Results:* We identified contextual factors, perceived risks, and perceived benefits as determinants of disclosing suicide loss. Contextual factors included social settings and characteristics of conversation partners. Perceived risks included emotional distress among oneself and others as well as stigma-related risks of disclosing. Perceived benefits included participants’ desire for authenticity and social support, as well as positive effects for grieving and fighting suicide stigma. *Limitations:* Findings are limited to the current female sample. *Conclusion:* SLS need support in identifying safe places for disclosure and in developing coping strategies to deal with suicide stigma and emotional distress experienced by themselves and others. Future research should investigate the general public’s ability and attitudes to provide support after suicide loss and investigate disclosure decisions among male SLS.

Suicide loss is common and often has a persisting and profound impact on those bereaved. Globally, every year, more than 700,000 people die by suicide (World Health Organization, 2021), and conservative estimates suggest that each suicide bereaves around 10 relatives and friends (Andriessen et al., 2017). Those numbers are concerning since suicide loss is associated with various mental health problems including suicidality (Pitman et al., 2014). Notably, in a UK study, one in 10 people bereaved by suicide subsequently attempted suicide themselves (Pitman et al., 2016). Interventions to improve mental health among suicide loss survivors [SLS, i.e., persons who lost a loved one by suicide and experienced at least moderate emotional distress (Feigelman et al., 2018)] are therefore needed.

Independent from the cause of death, the loss of a significant other is a painful but natural life experience. Despite initial intense grief-related distress, most bereaved persons have sufficient internal and external resources to cope with the loss and adjust to a life without the deceased (Lundorff et al., 2017). However, for some people, grief persists and causes significant impairment in daily functioning, a phenomenon referred to as complicated grief (Prigerson et al., 2009). Estimates suggest that around 10% of bereaved individuals are affected by complicated grief, which is associated with a broad range of health problems, including sleep disturbance, alcohol abuse, suicide, cardiovascular diseases, and cancer (Lundorff et al., 2017). While complicated grief can occur after any type of loss, it is more likely after losses that happen unexpectedly and particularly after loss by suicide (Tal et al., 2017). Nevertheless, coping with suicide loss is possible.

To prevent adverse mental health outcomes among SLS, it is important to identify modifiable determinants of healthy grieving among this group. According to the social–cognitive processing model, processing stressful life events largely depends on disclosing associated thoughts and feelings (Juth et al., 2015). Indeed, several studies found a positive link between disclosure and beneficial grieving outcomes among SLS (Levi-Belz et al., 2021). Moreover, keeping suicide loss secret was associated with negative mental health outcomes including suicidality (Oexle et al., 2020). However, many SLS struggle to share their experiences with others (Jordan, 2020). Furthermore, the beneficial effects of disclosure may depend on various factors, such as the relationship between teller and listener as well as experienced social reactions (Baddeley & Singer, 2009; Frey & Fulginiti, 2017). While initial evidence suggests that suicide stigma (i.e., stereotypes, prejudice, and discrimination toward persons affected by suicidality and/or their relatives) contributes to keeping suicide loss secret and hampers the beneficial effects of disclosure by resulting in negative social reactions (Hanschmidt et al., 2016), the determinants of disclosing suicide loss are poorly understood. We therefore conducted a qualitative study aiming to identify factors that influence disclosure decisions among SLS.

## Methods

### Research Team

The study was conducted by a diverse research team. NO (f) designed the study and wrote the manuscript, while NP (f) conducted and transcribed the interviews. Interviews were analyzed by NO, NP, and LM (f). NR (m) and NV (f) helped to finalize and interpret the findings. None of the team members had any kind of relationship with participants. Three members of the research team had experienced a loss by suicide in the past.

### Procedure

Between April 2019 and March 2020, we conducted individual interviews with a convenience sample of SLS in Germany. Eligible participants were at least 18 years old, had lost a significant other by suicide after their 14th birthday, and had experienced significant emotional distress after their loss, indicated by a score above 3 on a scale from 1 (*not at all*) to 5 (*extremely*) for the question “To what extent did you experience emotional distress after your loss by suicide?” (Feigelman et al., 2018) Persons who did not fulfill these criteria were excluded. Participants were recruited by (1) sending flyers to regional suicide loss self-help groups, (2) advertisements in regional newspapers, and (3) using snowball sampling (i.e., participating SLS were asked to distribute study information within their social networks). SLS could indicate their interest to participate via phone or e-mail. Study staff called interested persons to provide details about the study and conduct a brief screening interview. Then, written informed consent was obtained from those eligible and willing to participate. NP conducted interviews until reaching theoretical saturation. As this was an unfunded study, we chose a pragmatic approach to determine saturation. After each interview, the interviewer made notes about the most prominent themes, and no new themes were identified after 16 interviews; however, to be conservative, the remaining scheduled interviews were still conducted and analyzed. Interviews were conducted in-person (*n =* 7) or by telephone (*n =* 15), depending on participant preferences. The interviewer used a predeveloped semistructured interview guide (see Electronic Supplementary Material [ESM 1]) covering the following topics: (1) social interactions after suicide loss, (2) disclosing suicide loss, and (3) suicide stigma. The interview guide was pilot tested with one SLS, resulting in minor adaptations. All interviews took place in a private room at the university (if not by phone) and lasted 16–78 min (*M* = 37 min). Repeat interviews were not carried out. Participants received 20€ as compensation (plus travel costs). The study was approved by the Institutional Review Board of Ulm University (Reference: 73/19).

### Participants

In total, 32 persons contacted our team; 31 were eligible to participate, and 23 of these agreed to participate. One person who did not agree provided a reason (“emotionally not ready to talk about the loss”), while seven others could not be contacted and did not provide a reason. We initially interviewed 23 persons (22 women, 1 man); however, based on reviewer comments raising concerns regarding the lack of nonfemale representation, we excluded the one male participant and included 22 females in our analysis. Participants were on average 48 years old (range: 24–74 years). The time since loss ranged from 0 to 44 years (*M* = 12 years), and participants were on average 36 years old when they first experienced loss by suicide (range: 20–63 years). Most experienced one loss by suicide, but three participants reported two or three losses by suicide. For details on participant characteristics, see Table 1.

**Table 1:** Participant characteristics

Interview number	Age (years)	Relationship with the deceased person	Age at suicide loss (years)
01	40	Friend/son/cousin of husband	20/37/37
02	60	Son	45
03	29	Brother	27
04	26	Life partner	25
05	64	Husband	63
06	40	Cousin/godfather	36/39
07	54	Sister-in-law/life partner	51/53
08	24	Brother	23
09	64	Son	63
10	67	Son	63
11	74	Husband	30
12	51	Mother	21
13	26	Friend	21
14	53	Son	53
15	53	Brother	32
16	42	Father	31
17	54	Ex-husband	44
18	51	Mother	27
19	51	Father	48
20	58	Father	20
22	24	Grandfather	22
22	49	Mother	21

### Data Analysis

We audio-recorded interviews, transcribed them verbatim, and used qualitative content analysis (Mayring, 2014) within MAXQDA 12 to analyze the data. We used a set of deductive categories selected based on our research objectives (i.e., social reactions after suicide loss, positive/negative consequences of disclosing suicide loss, perceptions and experiences of suicide stigma) in addition to inductive categories. Three researchers (LM, NO, NP) independently sorted text items into deductive and inductive categories and met regularly to discuss unclear items and inductive codes. Six interviews were coded by two researchers, and discrepancies (not systematically recorded) were discussed until reaching consensus. After the analysis, we selected text items representing relevant themes and translated them from German to English focusing on the accurate reflection of their content rather than verbatim translation.

## Results

We identified three factors influencing disclosure decisions among SLS, namely context, perceived risks, and perceived benefits of disclosing suicide loss. Contextual aspects included social settings and characteristics of conversation partners. Perceived risks to disclose suicide loss included emotional distress among oneself and others as well as stigma-related risks of disclosing. Finally, perceived benefits included participants’ desire for authenticity and social support, as well as positive effects for healthy grieving and for fighting suicide stigma.

### Contextual Aspects

With regard to different social settings, participants were more hesitant to talk about their loss when they perceived their experiences to be irrelevant or inappropriate in certain situations. For example, some participants mentioned that they would not disclose suicide loss at their workplace for fear of negative consequences: “If I opened up at my new workplace, they would think less of me” (I13). Typically, participants shared their loss with family members and close friends as well as within religious settings and self-help groups. While participants used the internet to search for information about suicide and suicide loss, they rarely used online platforms to talk about suicide loss: “No, I never talked about it online (…), I don’t have enough trust for that” (I09). In addition to concerns about the trustworthiness of online settings, for some, online support was not available at the time of loss (i.e., those who experienced suicide loss before the widespread availability of the internet).

Disclosure decisions also depended on the presence of supportive conversation partners. It is important to note that many participants reported that their social network decreased after the loss: “I had friends […] that noticeably withdrew from me after the loss” (I01). Furthermore, even if potential conversation partners were available, most participants were quite selective in choosing whom to talk to. A general pre-requisite for disclosure was a close and trusting relationship: “They [conversation partners] must be trustworthy, because I don’t want them to gossip about it” (I17). To avoid hurtful reactions, participants only talked to others if they perceived them as good listeners (I06) who are tolerant (I07), empathic (I04), and emotionally ready (I17) to deal with the topic. One aspect that considerably facilitated disclosure and openness was shared experiences: “When someone made the same experience, you have something in common (…) and that makes a big difference” (I13).

### Perceived Risks of Disclosing Suicide Loss

Many participants stated that they were worried about causing emotional distress among both themselves and others when talking about their loss. In addition to unwanted emotional reactions such as crying (I19), they also mentioned potential long-lasting distress: “What prevents me from talking [about my suicide loss] is that I am worried, that it will distress me, that it will make me anxious again” (I14). Some participants mentioned that they do not want to impose themselves on others (I09) and bother them with their personal issues (I05), while most reported that talking about their suicide loss can be overwhelming for listeners: “I think most people are completely overwhelmed [when I disclose my suicide loss]. They cannot handle it” (I20). Consequently, participants worried that “conversations will die […] and relationships will suffer” (I28) when they talk openly about their loss and grieving experiences with others.

Participants reported several risks of disclosing suicide loss related to suicide stigma. In general, they described suicide as a topic that is generally very difficult to talk about: “Somehow it is still a taboo topic and people can’t deal with it” (I07). This was due to both a reluctance to disclose among SLS and reservations to respond to disclosure or actively address the issue among others: “Sometimes, I would really like to talk about it, but I usually get the feeling that no one wants to listen. No one is ever talking to me about my son” (I02). Many participants also mentioned fear of social labeling and associated devaluation when disclosing suicide loss. This included a general label avoidance [e.g., “I don’t always want to be seen as the one who lost her brother by suicide” (I08)] but also quite specific concerns such as being judged responsible for the death (I20), crazy (I06), or weak (I13). One woman said “There must be something off with someone who has a suicidal person on both sides of their family” (I06). Such fear of being judged negatively was particularly prominent among participants who lost their child or partner. Some participants did not only worry about being judged themselves but also wanted to protect their lost loved one from being devalued, e.g., as selfish (I13) or cowardly (I02): “It’s a violent mode of death and therefore it is difficult to talk about it. And I also want to protect my son’s privacy” (I02). Noteworthy, while all participants talked about fearing negative judgment, about a quarter actually experienced judgmental reactions.

### Perceived Benefits of Disclosing Suicide Loss

While all participants expressed their willingness to talk about their loss, some described a strong desire for honesty and authenticity: “I would feel like denying an important part of my life” (I17). Disclosure also facilitated healthy grieving. For example, one woman said that she “felt the need to constantly repeat what happened” (I15) to be able to accept the loss as part of her life. Furthermore, participants described disclosure as a necessity to receive social support and increase others’ thoughtfulness, e.g., to prevent hurtful situations such as people complaining about delayed trains due to a railway suicide (I06). For some participants, disclosure was a means to fight the taboo surrounding suicide: “I want people to realize that suicide is quite common, so that it is no longer taboo to talk about it” (I05). Finally, participants described a desire to support other SLS by sharing their experiences with suicide loss: “What makes disclosure easier for me is that it allows me to help others. To make them realize that there is a way out, that eventually life will go on” (I07).

## Discussion

Existing literature suggests that disclosure is an important aspect of healthy grieving after suicide loss (Levi-Belz et al., 2021; Oexle et al., 2020); however, SLS struggle to share their experiences with others, and the factors determining whether suicide loss is disclosed are poorly understood. Our findings suggest that disclosure decisions among SLS are complex and depend on the evaluation of perceived risks and benefits as well as the consideration of contextual aspects. According to the social interactional model of bereavement narrative disclosure, social relationships greatly determine whether loss is disclosed and whether disclosure is experienced as beneficial (Baddeley & Singer, 2009). Furthermore, as mentioned by our participants, certain social settings (e.g., workplace) discourage the disclosure of emotionally intense topics such as suicide loss. Additionally, participants reported restrained social relationships after their suicide loss, which was due to both their own social withdrawal but also social exclusion and unhelpful, often stigmatizing social reactions such as blaming (Mayer et al., 2022). Many SLS in our sample therefore struggled to identify trustworthy and supportive confidants. However, they described conversations with other SLS as extremely helpful, for example, within self-help settings, highlighting the need for close-knit self-help networks and peer support interventions. While online support programs for SLS are on the rise and have great potential to improve grieving outcomes among this group (Andriessen et al., 2019; Lestienne et al., 2021), SLS in our study mentioned that online settings did not seem trustworthy to them and thus were hesitant to share their loss experiences and seek support within online settings. As online support could reduce access barriers for SLS even in remote regions, future research should identify strategies to reduce current concerns regarding data safety and trustworthiness among SLS.

In line with previous studies (Hanschmidt et al., 2016; Shields et al., 2017), participants reported perceived risks of disclosing suicide loss related to suicide stigma. Specifically, they described suicide as a taboo topic that is associated with various stereotypes. Due to fearing labeling and negative judgment both toward themselves and their deceased loved one, many participants were hesitant to share their loss with others, a finding in line with one prior study (Maple et al., 2010). Interestingly, while almost all participants mentioned fearing stigmatizing reactions by others, fewer actually experienced such negative reactions. Furthermore, based on our data, disclosure decisions are also greatly inhibited by SLS’ fear to increase distress among both themselves and others.

Various previous studies suggested a positive link between disclosure and healthy grieving (Levi-Belz et al., 2021), which may at least partly be explained by disclosure potentially resulting in strengthened interpersonal relationships and social support (Baddeley & Singer, 2009). In line with that perspective, many participants in our study described disclosure as relieving and crucial for coping with loss. They chose disclosure to feel authentic, receive social support, and fight suicide stigma. Furthermore, some participants expressed their great desire to support other SLS by sharing their own grief experiences, highlighting the potential of peer support groups for the grieving process (Adshead & Runacres, 2022; Feigelman & Feigelman, 2008).

Despite providing important knowledge on determinants of disclosing suicide loss, several study limitations need to be considered. While we aimed for an equal gender distribution, we experienced great difficulties in recruiting male participants. Due to very small recruitment rates among men, we only included female participants in our analysis. A gender bias has been reported in disclosure of mental health problems with men being much less likely to seek help for mental health problems (Brown et al., 2019). Furthermore, grieving trajectories greatly vary by gender (Lundorff et al., 2020), and similar gender-based differences could exist in making disclosure decisions after suicide loss. Therefore, more research on male and also nonbinary experiences is needed. As previous research showed that disclosure decisions in general are influenced by cultural aspects (Ignatius & Kokkonen, 2007), our results might be limited to Germany or regions with similar cultural norms and values. While our findings suggest that social experiences after suicide loss could differ depending on one’s relationship to the deceased person (e.g., child, parent, friend), due to the chosen study design, a thorough investigation of such potential differences was not possible and should be the focus of future (quantitative) research. Furthermore, some participants experienced suicide loss more than 10 years ago, raising concerns about recall bias and the validity of findings. While for many SLS, having to make disclosure decisions is a permanent issue, societal and personal norms surely change over time, potentially resulting in quite different disclosure decisions and social reactions, which we were not able to investigate. Finally, while participants received a general summary of our research findings, their feedback on personal transcripts or findings was not systematically collected.

In summary, our findings suggest that disclosure decisions among SLS are complex and largely influenced by expected and experienced social reactions. While participants desired to be honest and authentic, many struggled to identify trustworthy confidants. SLS could therefore benefit from interventions to engage them with peers and support them in coping with suicide stigma and identifying trustworthy confidants for disclosure. One such potential intervention is the peer-led group program “Honest, Open, Proud” (HOP). HOP was initially developed to support disclosure decisions among persons with mental illness and was successfully adapted for several other stigmatized social groups such as people who survived a suicide attempt (Rüsch & Kösters, 2021; Scior et al., 2020). In a next step, HOP could be adapted and implemented for SLS aiming to support their disclosure decisions and therefore foster healthy grieving among this group. Within this context, it is important to note that disclosure risks described in this study are not merely theoretical fears but at least partly based on actual negative disclosure experiences (Mayer et al., 2022). Therefore, research investigating the ability and attitudes to provide social support after suicide loss among the general public is needed. Such research should focus on identifying modifiable factors to be targeted by interventions to improve attitudes and increase the competence of the general public in providing helpful support after suicide loss. Indeed, initial research suggests that beneficial outcomes of suicide loss disclosure could greatly depend on the quality of experienced social reactions (Baddeley & Singer, 2009; Frey & Fulginiti, 2017).

## Electronic Supplementary Material

The electronic supplementary material is available with the online version of the article at https://doi.org/10.1027/0227-5910/a000891

**ESM 1**. Interview guide.

## Notes

**Publication Ethics:** The study was approved by the Institutional Review Board of Ulm University (Reference: 73/19).
