# Supplementary material for: Perceived Determinants of Disclosing Suicide Loss: A Qualitative Study Among Women
Source: Crisis. 2022 Nov 29;44(6):470–6. doi: 10.1027/0227-5910/a000891 (PMC10658637; doi:10.1027/0227-5910/a000891)
Supplement: Supplementary file 1 [file cri_44_6_470_esm1.pdf]

https://doi.org/10.1027/0227-5910/a000891
© 2022 The Author(s). Distributed as a Hogrefe OpenMind article under the license CC BY 4.0 (https://creativecommons.org/licenses/by/4.0)

Perceived determinants of disclosing suicide loss: a qualitative study among women

Electronic Supplementary Material 1

Interview Guide

Introductory question:

Can you please tell me more about your loss by suicide, for example who you lost and when the suicide happened?

Social interactions after suicide loss:

- How did others react when they learned about your loss by suicide?
  - Positive/negative reactions?
  - Did reactions differ, when people knew the type of death?
  - Did people's reactions change over time?
- Can you describe how other people supported you in your grieving process?
  - What type of support was helpful/not helpful?
  - Did you receive professional support? If yes, was this helpful?
  - What factors made it easier/harder for you to receive professional or social support?

Disclosing suicide loss:

- How did you feel when telling others about your loss by suicide?
  - How did you feel while you were talking to others and how did you feel afterwards?
  - What factors make it easier/harder for you to talk about your loss by suicide?
  - When you talk about your loss by suicide, were there certain aspects you keep secret?
  - What are the advantages/disadvantages to talk about loss by suicide?
  - What are the advantages/disadvantages to keep loss by suicide secret?
  - With whom do you currently talk about your loss by suicide?
  - Do you use social media/online settings to talk about your loss by suicide?

Suicide stigma:

- When you talked about your loss by suicide, did you get the impression that other people judged your loved one negatively? If yes, how did that make you feel?
- When you talked about your loss by suicide, did you get the impression that other people judged you negatively? If yes, how did that make you feel?
- Due to your loss by suicide, did you ever judge yourself negatively?

Closing question:

- Is there anything else that you would like to talk about regarding your experiences after losing a loved one by suicide?
